# Gut Microbiota Modulation by Dietary Barley Malt Melanoidins †

**DOI:** 10.3390/nu12010241

**Published:** 2020-01-17

**Authors:** Nesreen Aljahdali, Pascale Gadonna-Widehem, Pauline M. Anton, Franck Carbonero

**Affiliations:** 1 Department of Biological Science, King Abdulaziz University, Jeddah 21551, Saudi Arabia; nhaljahdali@kau.edu.sa; 2 Cell and Molecular Biology Program, University of Arkansas, Fayetteville, AR 72701, USA; 3 Transformations et Agro-Ressources—EA7519-Institut Polytechnique UniLaSalle, 60026 Beauvais, France; gadonna.pascale@gmail.com (P.G.-W.); pauline.anton@unilasalle.fr (P.M.A.); 4 Department of Food Science, University of Arkansas, Fayetteville, AR 72704, USA; 5 Department of Nutrition and Excercise Physiology, Elson Floyd School of Medicine, Washington State University-Spokane, 412 East Spokane Falls Boulevard, Spokane, WA 99202, USA

**Keywords:** Maillard reaction products, melanoidins, short-chain fatty acids, gut microbiota, prebiotic

## Abstract

Melanoidins are the final Maillard reaction products (protein–carbohydrate complexes) produced in food by prolonged and intense heating. We assessed the impact of the consumption of melanoidins from barley malts on gut microbiota. Seventy-five mice were assigned into five groups, where the control group consumed a non-melanoidin malt diet, and other groups received melanoidin-rich malts in increments of 25% up to 100% melanoidin malts. Feces were sampled at days 0, 1, 2, 3, 7, 14, and 21 and the microbiota was determined using V4 bacterial 16S rRNA amplicon sequencing and short-chain fatty acids (SCFA) by gas chromatography. Increased melanoidins was found to result in significantly divergent gut microbiota profiles and supported sustained SCFA production. The relative abundance of *Dorea*, *Oscillibacter*, and *Alisitpes* were decreased, while *Lactobacillus*, *Parasutterella*, *Akkermansia*, *Bifidobacterium*, and *Barnesiella* increased. *Bifidobacterium* spp. and *Akkermansia* spp. were significantly increased in mice consuming the highest melanoidin amounts, suggesting remarkable prebiotic potential.

## 1. Introduction

The Maillard reaction (MR) generates several low-weight molecules by reducing sugars and amino acids during food thermal processing and storage, such as Amadori rearrangement products, furfural, reductones, and other dicarbonyl compounds. These low-weight compounds are often recombined through a range of advanced MR to form melanoidins [1], which are the final products of the MR [2]. Melanoidins are brownish, heterogonous, insoluble molecules, and were traditionally considered to be high-molecular weight (HMW) molecules [3], but recent reports have shown that melanoidins also include some low-molecular weight (LMW) members [4]. Melanoidins produced in foods are predominantly HMW, and the molecular weight of melanoidins is directly correlated with heating intensity and time [5]. For example, the average molecular weight (MW) of unroasted malts is <10 kilodalton (kDa), whereas that of roasted malts is around 320 kDa [6]. The chemical structures of melanoidins are complex and difficult to determine, but the concentrations of sugars and amino acids in roasted barley have been identified [7]. During the roasting of barley, significant increases in total sugar, dextrin, and melanoidins were detected, while hemicellulose and starch significantly decreased [7]. The average molecular weight of roasted malt melanoidins was 320 kDa [6]. 

In contrast with other Maillard reaction products (MRP), melanoidins are generally considered to be harmless, and even potentially beneficial to human health [8]. Though some studies reported that dietary melanoidins might display moderate genotoxicity and cytotoxicity effects [9,10], several studies reported potential health benefits, including antioxidant, antihypertensive, antimicrobial, and prebiotic properties of food melanoidins [11,12]. Data from metabolic transit studies showed that melanoidins can escape digestion and pass into the upper gastrointestinal tract (GIT), where they are likely subject to fermentation by resident gut microbes [13,14,15]. To illustrate, only 27% of the LMW constituents of melanoidin products were absorbed in the intestines, and only 4.3% of the HMW melanoidins were excreted in feces and urine [9]. Fecal excretion of bread melanoidins was shown via a new method for the quantification of soluble melanoidins [16], confirming that gut microbiota and melanoidins interact [17].

It was actually suggested that dietary melanoidins possess prebiotic properties [5] due to their structural similarities with fibers [18]. Interestingly, studies on the interactions between gut microbiota and melanoidins initially focused on antimicrobial activity, predominantly in batch cultures [19,20,21]. For instance, data from in vitro and in vivo studies showed that melanoidins suppressed *Helicobacter pylori* infection [22]. Moreover, melanoidins were shown to kill *Escherichia coli* by causing irreversible changes to both the inner and outer membranes [23]. While knowledge on the role of the gut microbiota in non-digestible polysaccharides and fiber fermentation is extensive [24,25], gut microbiota fermentation of MRPs has been scarcely studied [26], with even less knowledge on melanoidins [27]. In an in vitro study, melanoidins were shown to increase the growth of gut anaerobes during mixed culture growth [28]. *Bifidobacteria* strains were shown to use bread melanoidins as a carbon source in batch cultures [27], while coffee melanoidins increased the number of anaerobic bacteria belonging to *Bacteroides* and *Prevotella* during fermentation in an in vitro study [29]. Another food rich in melanoidins is beer because melanoidins are present in malts, with HMW melanoidins being are more abundant in kilned malts [30]; this source of melanoidins has not yet been assessed for its effect on the gut microbiota. The objective of this study was to determine the impact of long-term consumption of increasing melanoidin concentrations from barley malts on the gut microbiota and fermentation patterns of healthy mice.

## 2. Material and Methods

### 2.1. Experimental Animals

The animal study was conducted at the Animal House Facility of the University of Arkansas after receiving approval from the Institutional Animal Care and Use Committee (IACUC). Seventy-five male mice (Mus musculus strain C57BL/6J) aged 8 weeks (20 g) were purchased from Jackson Laboratory (Farmington, USA). Mice were housed in stainless steel cages under a controlled temperature (70 °F) and a 12 h light–dark cycle, with free access to water and food. Before dietary intervention, mice were provided with Teklad (standard) 40 g of chow pellets (Envigo, Madison, WI, USA).

### 2.2. Experimental Design

Melanoidin-free and melanoidin-rich barley malts were purchased from Weyermann Company (Northern Brewer, USA). Melanoidin-free malts were considered to be normal barley grains pre-germinated to release saccharolytic and amylolytic enzymes for beer-brewing purposes and contained low amounts of LMW melanoidins (Briess 2-row Malt). Melanoidin-rich malts were considered to be enriched in HMW melanoidins due to intense toasting (Weyermann^®^ Melanoidin), resulting in grain-browning and specific organoleptic properties [7,31]. The mice were assigned to receive 40 g of melanoidin malts in the first week. The portion of malts was increased by 20 g each consecutive week [25]. Five groups of 15 mice each were each assigned to a different treatment. Among each group, five groups of three mice were assigned to different cages and each cage was considered as a replicate (*n* = 5 for each group). Cages were randomized in the rack to limit potential block effects.

Group (1): Melanoidin-free malts (0% melanoidins) only.

Group (2): 75% of melanoidin-free malts and 25% of melanoidin-rich malts.

Group (3): 50% of melanoidin-free malts and 50% of melanoidin-rich malts

Group (4): 25% of melanoidin-free malts and 75% of melanoidin-rich malts

Group (5): Melanoidin-rich malts (100% melanoidins) only.

Malts were added to the stainless cages twice a week, and the unconsumed amounts were measured before adding new malts. Body weight was measured at 7, 18, and 25 days. The mice were transferred from stainless steel cages to metabolic cages (Tecniplast Cdd, 170013) for 6 h/day in order to collect feces at day 0, 1, 2, 3, 7, 14, and 21. Day 0 samples represented the baseline, with all groups previously fed chow pellets. After that, each group was provided with their specific amounts of melanoidin malts over the 21 days.

### 2.3. Fecal Short Chain Fatty Acids (SCFAs) Quantification

Short chain fatty acids (SCFAs), specifically, acetate, propionate, and butyrate, were measured by gas chromatography (GC) for the 0% and 100% groups. Briefly, 1 g of fecal sample was transferred into centrifuge tubes, and 9 mL of distilled water was added. After vortexing and centrifugation, 900 μL of the supernatant was transferred into 2 mL tubes containing 100 μL of buffer consisting of the internal standard 4-methyl-valeric acid (50 mM), meta-phosphoric acid (50%), and copper sulfate (1.56 mg/mL). After vortexing and centrifugation, 1 μL of sample was loaded to the GC. SCFA concentrations were estimated by the integration of peak areas in relation to acetate, propionate, and butyrate standards (Sigma-Aldrich, Germany) [32].

### 2.4. DNA Extraction and PCR Amplification

Genomic DNA was extracted from mice fecal samples using commercial QIAamp DNA stool Mini Kit (Qiagen, Germany) following the manufacturer’s protocol, with the addition of bead-beating at 5 m/s for 60 s, which was performed twice [33]. PCR amplifications were performed in 25 μL reactions with 1 μL of DNA template, 2 μL of universal primer (8F and 1541R), and 22 μL of KAPA HiFi mastermix (KAPA Biosystems, Wilmington, MA, USA), followed by agarose gel electrophoresis by using 1 μL of SYBR-safe DNA Gel Stain (Thermo Fisher Scientific, Wilmington, MA, USA) fluorescent dye to confirm the success of the PCR. The PCR index was accomplished by targeting the V4 region of the bacterial 16S rRNA gene [34]. Briefly, the PCR dual-indexed strategy was performed in 27 μL reaction components with 2 μL of DNA template, 2 μL of index primers, and 23 μL of AccuPrime™ Taq DNA polymerase (Invitrogen, Wilmington, MA, USA) following the manufacture’s protocol. Amplifications were performed by initial denaturation at 95 °C for 3 min, followed by 25 cycles of denaturation at 95 °C for 30 s, primer annealing at 55 °C for 30 s, and extension at 72 °C for 1 min.

### 2.5. Library Preparation and Sequencing

Illumina MiSeq sequencing was used to study the composition of gut microbiota by targeting the V4 region of the bacterial 16S ribosome RNA gene of each group following the dual-indexed strategy [34]. Normalization of the PCR products was completed to elute short primers, unincorporated dNTPs, enzymes, short-failed PCR products, and salts from PCR reactions using Invitrogen SequalPrep kits, following the manufacturer’s protocol. Q-PCR was performed using the PerfeCta NGS library quantification kits (Quanta Biosciences, USA), following the manufacturer’s protocol. Quality checks were also performed on a Tape-Station 2100 (Agilent, Santa Clara, CA, USA) to provide the exact sizes of the DNA, which were 394 base pairs and 424 base pairs.

The libraries were pooled, denatured with NaOH, and diluted to 0.75 nM, following recommended Illumina protocols. The pooled denatured libraries were diluted to 6 pm as a final concentration, with the addition of 20 pm of Phix V3. The diluted denatured libraries were loaded onto an Illumina MiSeq sequencing cartridge V3-600 cycles, with the addition of 3 sequencing primers due to the use of different indices, as described by Kozich et al. [34]. The runs were monitored with Sequence Analysis Viewer with particular emphasis on appropriate cluster density (700–800 k/mm^2^) and quality scores (final >Q30 score of >70%).

From 175 samples, a total of 10,123,928 raw sequences were obtained, of which 9,290,708 high-quality reads were used for further analysis. Samples yielding less than 3000 high-quality reads (*n* = 9) were discarded; the remaining samples analyzed had an average of 502,020 ± 37,097 reads per sample. All reads were analyzed together using the MOTHUR1.39.5 pipeline.

### 2.6. Bioinformatics and Statistical Analyses

FASTQ files were readily demultiplexed by the built-in BaseSpace Sequence Hub program and downloaded from the BaseSpace website. The 16S amplicon reads were analyzed by using the MOTHUR software package 1.39.5 following the Illumina Standard Operating Procedures (SOP) [35] (https://www.mothur.org/wiki/MiSeq_SOP). Briefly, sequences were screened and aligned to the Silva database (Release 128) for the 16S RNA gene sequences. Subsequently, operational taxonomic units (OTUs) were picked and assigned to taxonomic groups. The resulting OTUs and taxonomic tables were exported to Excel sheets for basic analysis.

The results of feed intake, average daily gain, and short-chain fatty acids were analyzed by analysis of variance (ANOVA) followed by Tukey’s post hoc test, with *p* < 0.05 considered to show significant difference between groups. The results of the microbiota counts were analyzed by the Kruskal–Wallis and Mann–Whitney pairwise tests, with *p* < 0.05 considered to show significant difference between groups, and by non-metric multidimensional scaling (NMDS) based on count-distance metrics (Bray–Curtis similarity index; Analysis of Similarities (ANOSIM): *p* < 0.05) considered to show significant similarities between groups using Past3 software (version 2.17, Øyvind Hammer, University of Oslo, Oslo, Norway) [36].

## 3. Results

### 3.1. Impact of Diet on Feed Intake and Average Daily Gain

Feed intake varied considerably during the study for all mice. A significant increase was observed between 4 and 7 day, followed by a return to the baseline feed intake afterwards. Intriguingly, feed intake decreased significantly after day 18 (Table 1). Dietary treatments had little impact on feed intake, with only a slight (but significant) increase of feed intake for the 25% group (0.54 ± 0.02 g/day) relative to the groups that consumed 0% and 100% melanoidin malts (0.48 ± 0.02 g/day and 0.44 ± 0.02 g/day).

Average daily weight gain (ADG) varied during the study (*p* < 0.05; Table 2). The 0% and 25% groups exhibited significantly lower ADG during the first seven days, and significantly higher ADG in the last seven days. The 50% and 75% groups had completely inverse dynamics in ADG (Table 2). The 100% group maintained relatively low ADG throughout the study.

### 3.2. SCFAs Abundance and Dynamics

The quantity of SCFAs measured in the feces from the 0% and 100% groups are shown in Table 3 from day 0 to day 21. Overall, the total SCFA concentrations decreased markedly in the mice that received 0%, but remained stable in the mice fed 100% melanoidin malts over the experiment. There were no apparent differences in the proportions of acetate in the mice fed 0% and 100% at days 0, 3, 7, and 14. However, there were significant differences in acetate between the mice that received 0% melanoidin malts and the mice that consumed 100% of melanoidin malts at day 21 (*p* < 0.05; Table 3). Consumption of melanoidins maintained acetate production at stable levels, while consumption of non-melanoidin malts resulted in a significant decrease. Significant decreases in the quantity of propionate were observed in mice fed 0% at days 14 and 21 and in mice that consumed 100% of melanoidin malts at days 7, 14, and 21. The amount of propionate between the 0% and 100% melanoidin malt groups was significantly different at day 21 (*p* < 0.05; Table 3); melanoidin malt consumption also allowed for maintenance of higher propionate production. Significant decreases in the amount of butyrate were observed in mice fed 0% at days 14 and 21 and in mice that consumed 100% of melanoidin malts at days 3, 7, 14, and 21. The quantity of butyrate between the 0% and 100% melanoidin malt groups was significantly different at day 14 (*p* < 0.05; Table 3).

### 3.3. Impact of Diet on Gut Microbiota Profiles and Dynamics

**Figure 1:**
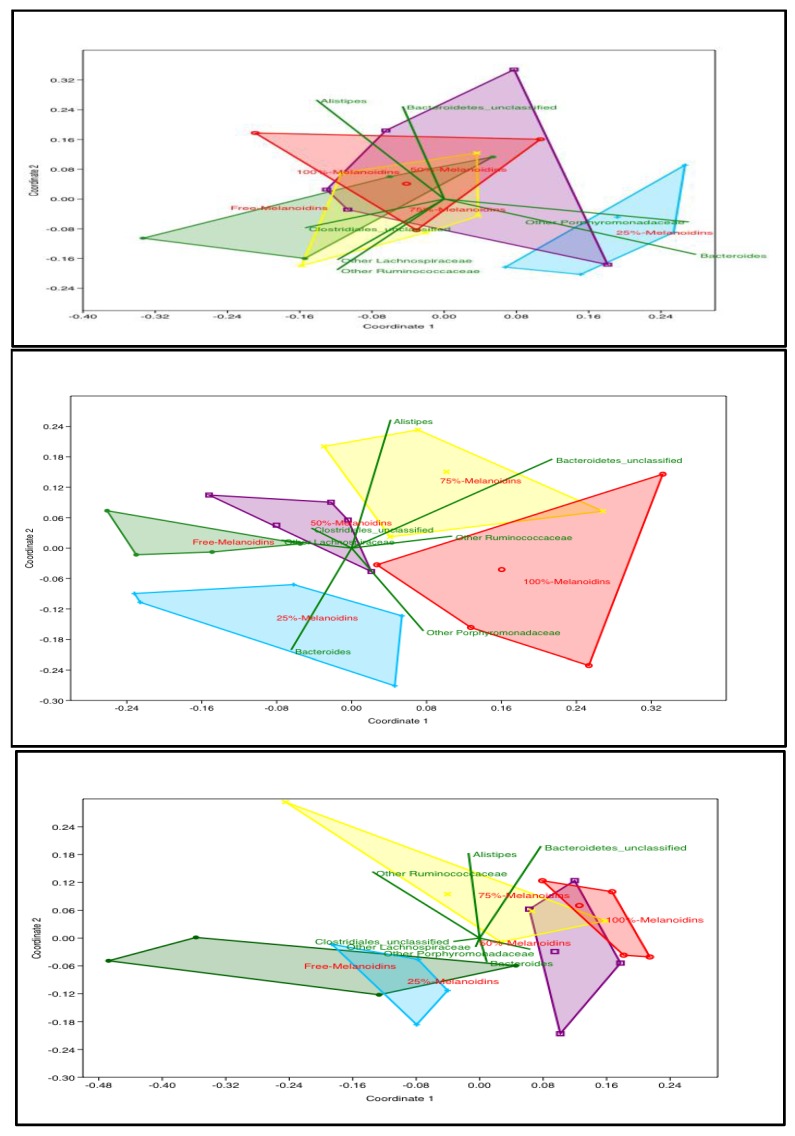
Impact of increasing dietary melanoidin malts on the composition of the gut microbiota (non-metric multidimensional scaling (NMDS)): Day 0 showed no significant difference (ANOSIM *p* > 0.05); day 7 showed the 25%, 50%, 75%, and 100% melanoidin malt groups to be significantly different from the 0% group (ANOSIM *p* < 0.05); day 21 showed the 0% and 25% groups to be significantly different from 50%, 75%, and 100% groups (*n* = 5).

The NMDS plots for days 0, 3, 7, 14, and 21 showed that the gut microbiota profiles of the five groups became distinctly different at days 3, 7, 14, and 21 (ANOSIM *p* < 0.05), while they were not distinguishable on day 0 (Figure 1). On day 3, the groups that consumed higher percentages of melanoidin malts (75% and 100%) were significantly different from the other groups (0% and 25%), while the 50% group was not significantly different from any group (Supplementary Figure S1; ANOSIM *p* < 0.05). On day 7, the groups that consumed melanoidin malts (25%, 50%, 75%, and 100%) were all significantly different from the group that consumed 0% melanoidin malts (Figure 1; ANOSIM *p* < 0.05). On day 14, the 75% and 100% groups clustered significantly and separately from the 0% and 25% groups (Supplementary Figure S1; ANOSIM *p* < 0.05). Long-term (day 21) consumption of melanoidins resulted in significant clustering, separating the 50%, 75%, and 100% groups from the 0% and 25% groups (Figure 1; ANOSIM *p* < 0.05).

### 3.4. Impact of Melanoidin Malts on the Composition of the Gut Microbiota

**Figure 2:**
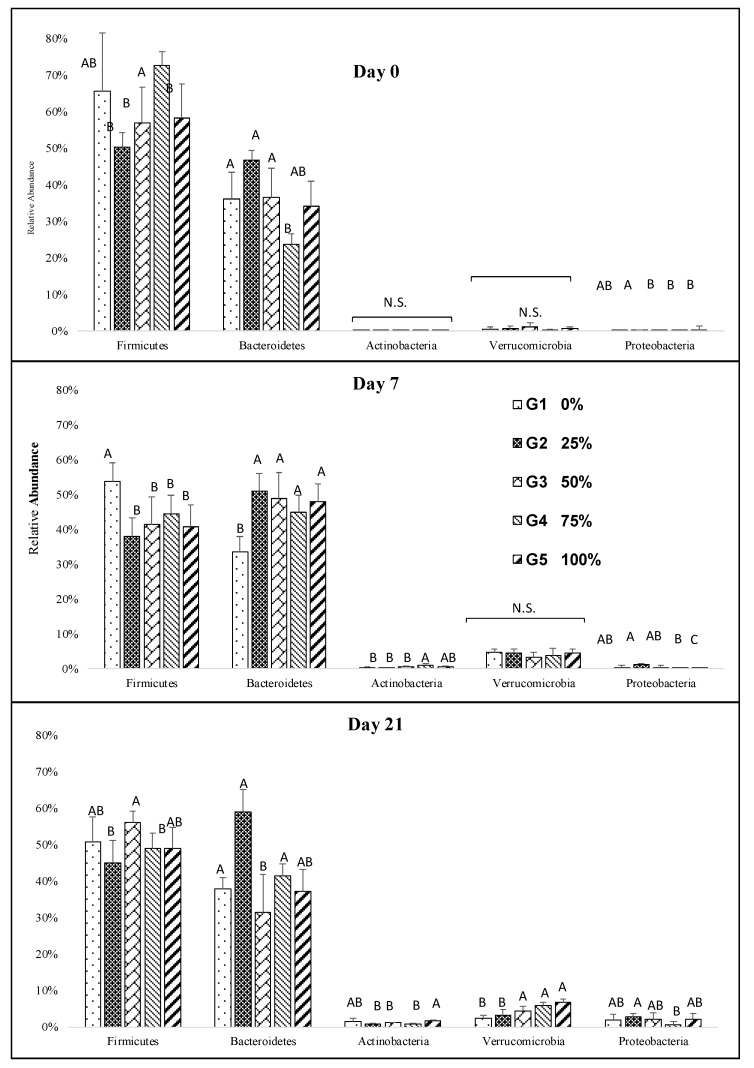
Impact of increasing dietary melanoidin malts on the composition of the gut microbiota at the phylum level during the study. Significant differences (*p* < 0.005) are indicated by different letters (*n* = 5).

Overall, the most abundant phyla detected were Firmicutes, Bacteroidetes, Actinobacteria, Verrucomicrobia, and Proteobacteria. The consumption of any combination of malts resulted in a significant decrease in Firmicutes. Conversely, malt consumption resulted in a distinctive increase in Bacteroidetes, Actinobacteria, Verrucomicrobia, and Proteobacteria during the study (Figure 2). Although there were no or slight differences between the groups for the three most abundant phyla, long-term melanoidin consumption resulted in a significant increase in Actinobacteria in the groups that received 100% melanoidin malts (Figure 2). Increased abundance of Verrucomicrobia was observed in the groups fed 50%, 75%, and 100% melanoidin malts at day 21 (Figure 2).

Regardless of treatment, the abundances of *Dorea*, *Oscillibacter*, and *Alisitpes* were decreased, but the relative abundances of *Lactobacillus*, *Paresutterella*, *Akkermansia*, *Bifidobacterium*, and *Barnesilla* were increased during the study (Supplementary Figure S2). In addition, there were no significant differences in any genera at day 0 between the groups, except that the 25% group had significantly higher *Bacteroides* and *Parasutterella* and lower *Alistipes.*

**Figure 3:**
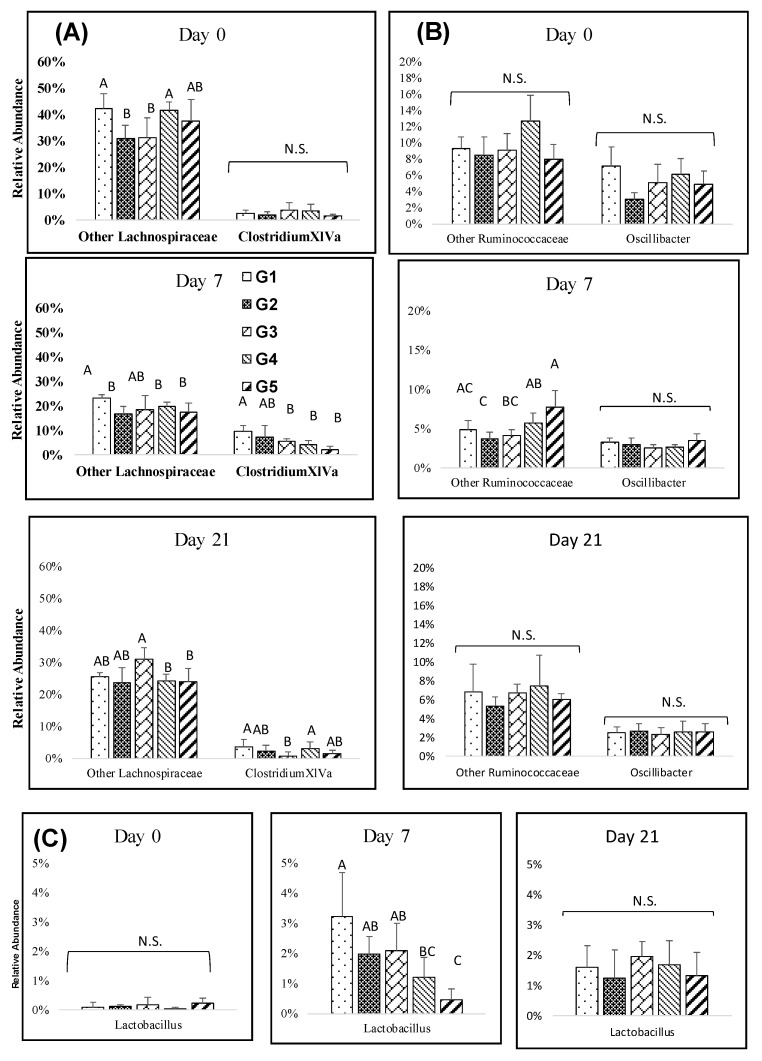
Impact of melanoidin malts on responsive genera relative abundances among the Firmicutes. (**A**) Lachnospiraceae, (**B**) Ruminococcaceae, and (**C**) Lactobacillaceae. Significant differences (*p* < 0.005) are indicated by different letters (*n* = 5).

Several genera among the Firmicutes were found to be significantly affected by the amount of melanoidins from day 3 to day 21. The relative abundance of *Clostridium XIVa* generally increased at days 3 and 7, with significantly lower abundances in high melanoidins groups, but the differences decreased over the long-term (Figure 3A). The abundance of *Dorea* was higher on day 0, but after melanoidin malt consumption, *Dorea* decreased and resulted in slight or non-significant differences between the groups at days 7, and 21 (Supplementary Figure S3B). *Clostridium XIVb, Roseburia,* and *Lactobacillus* resulted in gradual significant increases observed at days 7, and 21. Lower abundances of *Clostridium XIVb* and *Lactobacillus* were found in the high melanoidin groups compared to other groups at days 7 (Figure 3B,C). The relative abundance of *Oscillibacter* decreased at days 7, and 21, but there were no significant differences between the groups (Figure 3B). 

**Figure 4:**
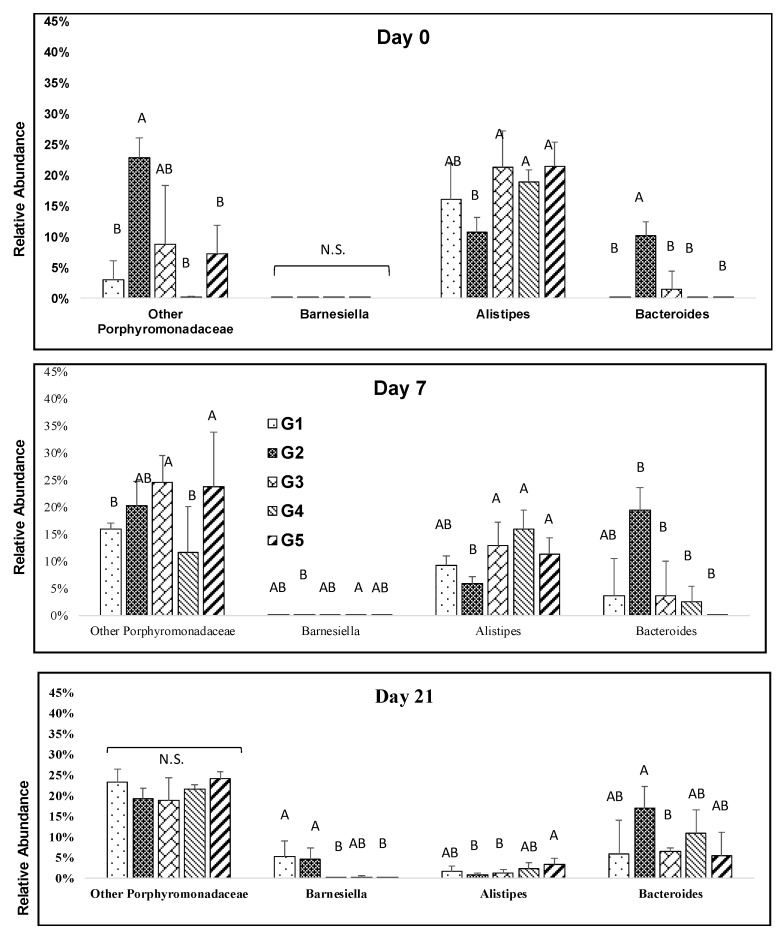
Impact of melanoidin malts on responsive genera relative abundances among the Bacteroidetes. Significant differences (*p* < 0.005) are indicated by different letters (*n* = 5).

The melanoidin malt consumption resulted in significant increases in *Barnesiella* (Bacteroidetes) at days 7, and 21. *Barnesiella* abundance was significantly higher in low percentage melanoidin malts (Figure 4). The relative abundance of *Alistipes* was significantly depleted during the study at days 7, and 21, although the abundances were slightly different among groups (Figure 4). The relative abundance of *Bacteroides* resulted in an increase during the study and a sharp increase was observed in the 25% group (Figure 4).

**Figure 5:**
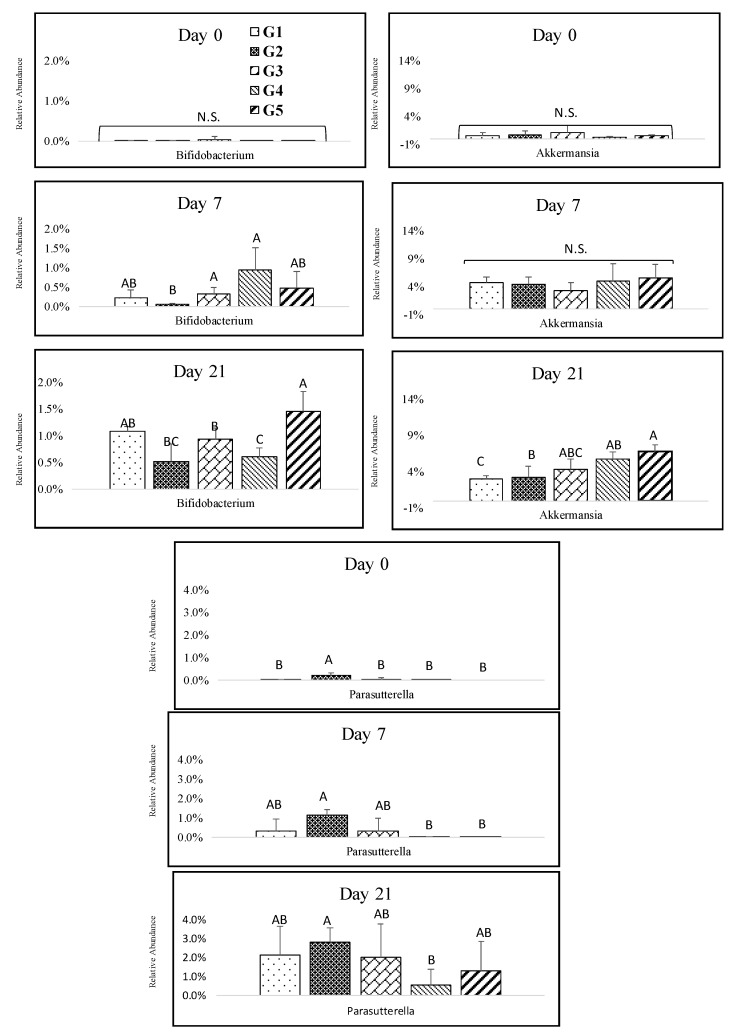
Impact of melanoidin malts on responsive genera relative abundances among the other phyla. Actinobacteria (*Bifidobacterium*), Verrumicrobia (*Akkermansia*), and Proteobacteria (*Parasutturella*). Significant differences (*p* < 0.005) are indicated by different letters (*n* = 5).

The abundant genus of Actinobacteria was *Bifidobacterium,* which increased throughout the study with a significantly higher abundance for the 100% melanoidin malt consumption group (Figure 5). The consumption of melanoidin malts resulted in a gradual increase in *Akkermansia* at days 3, 7, 14, and 21. There was a significant difference between the groups at day21, and the high abundance of *Akkermansia* was in the group that consumed 100% of melanoidin malts (Figure 5). The responsive genus among Proteobacteria was *Parasutterella*, which exhibited a sharp significant increase throughout the study, but we observed only slight differences between the groups (Figure 5).

The relative abundance of several Firmicutes genera (*Clostridium XIVb* and *Lactobacillus*), as well as *Bifidobacterium* (Supplementary Figure S3) and *Akkermansia* (Supplementary Figure S3) were increased significantly in all groups that consumed 0%, 25%, 50%, 75%, and 100% of melanoidin malts throughout the study from day 0 to day 21, and there were slight or significant differences among the days (*p* < 0.005). Significant increases in *Parasutterella* were present in the mice that were fed 0%, 25%, and 50% melanoidin malts during the study, but the relative abundance of *Parasutterella* was lower in groups that received 75% and 100% melanoidin malts throughout the study from day 0 to day 2, and there were no or only slight differences among the days Supplementary Figure S3). However, the relative abundance of *Dorea* (Firmicutes; Supplementary Figure S2) and *Alistipes* (Bacteroidetes; Supplementary Figure S3) decreased gradually through the study from 0 day to 21 day in all mice that were fed 0%, 25%, 50%, 75%, and 100% melanoidin malts, and there were significant differences between the days (*p* < 0.005).

## 4. Discussion and Conclusions

The purpose of this study was to investigate the impact of melanoidin-rich malts, which may represent a major source of specific dietary melanoidins for humans, on the composition of the gut microbiota and their potential prebiotic effects. It was reported that a large proportion of HMW melanoidins are excreted in feces and urine [13]. We noted the brownish color of the feces and urine from mice that were fed 100% melanoidin malts compared to mice that consumed 0% melanoidin malts after 21 days in this study. Several studies reported that dietary melanoidins could escape digestion and pass through the gastrointestinal tract, where they may be fermented by the intestinal microbiota; and dietary melanoidins were suggested to behave like dietary fiber by enhancing the growth of beneficial gut bacteria [13,18,37,38].

The consumption of coffee melanoidins had no effect on the weight gain of rats that were fed a high-fat diet [39]. However, the consumption of germinated barley (malt) resulted in a significant decrease in body weight in mice [40]. In this study, we found that the average daily gain initially increased but was subsequently lowered by the consumption of melanoidin-rich malts, confirming that dietary melanoidins have a limited impact on weight gain.

The quantification of SCFAs in feces is a useful index of the fermentative potential of the gut microbiota. In this study, the proportion of acetate was stable throughout, but the quantities of propionic and butyrate decreased. The concentrations of butyrate, acetate, and propionate were higher in the mice that consumed 100% melanoidin malts compared to 0% melanoidin malts at days 14 and 21, respectively. The total SCFA concentrations were stable in mice that consumed 100% of melanoidin malts, while a significant decrease was observed in the control group, which was consistent with the higher SCFA concentration observed in rats that were fed bread crusts compared to the controls [41]. Importantly, acetate was mainly responsive to malt melanoidin consumption, whereas acetate was the increased SCFA in the bread crust consumption trial [41]. These results were in favor of the hypothesis that melanoidins modulate the gut microbiome and fermentation patterns in a similar fashion to dietary fibers or other complex polysaccharides. While whole-grain barley is considered an excellent source of dietary fiber, milling and malting considerably decreased the amount of fiber in barley malts, thereby explaining the drop in SCFA production. It appears that roasting may represent a simple approach to restore the presence of fermentable compounds, namely melanoidins.

In this study, the relative abundance of Firmicutes decreased, but Bacteroidetes, Verrucomicrobia Acinobacteria, and Proteobacteria increased during the study, which was consistent with a previous study that included higher abundances of Verrucomicrobia and Acinobacteria and a lower abundance of Firmicutes in rats fed barley malt [25]. Zhong et al. found that consumption of whole-grain barley resulted in the increase of *Akkermansia* spp. and *Ruminococcus* spp., while *Roseburia* spp. and *Lactobacillus* spp. were more abundant in the cecum of rats fed barley malt, compared to the control group that was enriched in *Oscillospira* spp. and *Dorea* spp. [25]. We observed that *Ruminococcus* spp. and *Lactobacillus* spp. had higher abundances in mice fed 0% melanoidin malts. However, there were significant decreases in *Dorea* and *Oscillibacter* throughout the study, which may be in line with a previous study showing the reduction of some genera belonging to Firmicutes, such as *Dorea,* after oral supplementation of glutamine [42]. A significant increase in *Roseburia* spp. was observed during the study, which may be in line with a study on *Roseburia* growth in healthy humans who consumed whole-grain barley for 60 days [43]. The effects of dietary fiber sources in an alfalfa diet showed an increase in *Clostridium* cluster *XIVb* compared to the pure cellulose diet of suckling piglets [44]. We indeed observed increases in *Clostridium* cluster *XIVb* during this study, with slight differences between the groups.

In this study, we observed a significant increase in *Bacteroides* spp. in mice that were fed fewer melanoidin malts, especially 25% melanoidin malts, which may be in line with the previous in vitro report of an increase in the proportion of *Bacteroides* spp. in light- and medium-roasted coffee compared to dark-roasted coffee [29]. *Bacteroides* spp. are known for their ability to ferment different mucin polysaccharides because they possess a wide range of carbohydrate-depolymerizing enzymes [45]. Moreover, in the present study, we observed significant differences in average daily gain and the proportion of consumed found in mice that consumed 25%, which had higher abundances of *Bacteroides* spp. Relative abundances of *Barnesiella* spp. were found at low levels, which made up less than 1% of an individual’s total gut bacteria; they, in particular, are known for their ability to control the spread of highly antibiotic-resistant bacteria [46,47]. Significant decreases in *Barnesiella* were detected in a guinea pig model fed a Western diet associated with metabolic syndrome [48]. However, dietary-resistant starch resulted in significant increases in *Barnesiella*, *Ruminococcus*, and *Bifidobacterium* in a rodent colitis-associated colorectal cancer model, which suggested resistant starch might have a beneficial effect on patients with ulcerative colitis [49]. In the present study, significant increases in *Barnesiella* were observed, especially in the mice fed 0% and 25% at 21 days. A decreased abundance of *Alistipes* was also shown in the present study. Wang et al. similarly showed that oligosaccharide treatment decreased the levels of *Alistipes* in mice with constipation [50].

*Akkermansia* spp. are known as mucin-degrading bacteria that use glycated proteins as an energy source [51]. *Akkermansia muciniphila* are known for their ability of anti-inflammatory effects in the intestine. A significant decrease in *A. muciniphila* was found in colitic mice [52]. We detected a high abundance of *Akkermansia* spp. in mice that were fed melanoidin-rich malts. *Bifidobacterium* spp. were also found in high abundances in the melanoidin-rich malt group. Dietary fiber enhances the growth of *Bifidobacterium* spp. [27]. Coffee consists of soluble fiber, mainly galactomannans and arabinogalactans [29]. The roasted coffee silverskin, which contains 60% of the total dietary fiber, enhanced preferential growth of *Bifidobacterium* spp. in vitro compared to other anaerobic bacteria [53]. An increase in the population of *Bifidobacterium* spp. was also shown after coffee consumption in humans [54]. In addition to coffee, bread crust melanoidins promoted the growth of *Bifidobacterium* spp. using a static batch culture [27]. The type of melanoidins plays an important role in enhancing the growth of *Bifidobacterium* spp. Coffee melanoidins are characterized by considerable carbohydrates, but bread crust melanoidins demonstrate a prevalence of amino acids. Thus, the coffee melanoidins increased the growth of *Bifidobacterium* spp. compared to bread crust melanoidins [27]. The structures of melanoidin malts are similar to coffee melanoidins in regard to their considerable amount of carbohydrates and fibers. Furthermore, distinct increases in *Parasutterella,* known as the saccharolytic strain, were detected during the study, which might be in line with previous reports of the proportion of *Parasutterella* that were elevated by carbohydrate consumption in rodent models [55].

We conclude that the long-term consumption of melanoidin malts increased microorganisms often considered beneficial, such as *Bifidobacterium*, *Akkermansia*, and *Lactobacillus,* although there were no significant differences in the population of *Lactobacillus* between the groups that consumed 0% and 100% melanoidin malts. These results confirm that the gut microbiota responds differently to different melanoidin-rich foods, and that melanoidin-rich malts appear to exert potentially beneficial changes, a property that could potentially lead to the development of novel prebiotic foods.

## Figures and Tables

**Table 1:** Temporal variation of feed intake for all mice considered as one group (*n* = 75). Data are expressed as mean ± SEM.

Feed Intake by Days (g/day)	Estimated Mean
4 days	0.53 ± 0.01 ^b^
7 days	0.74 ± 0.01 ^a^
11 days	0.46 ± 0.01 ^cd^
14 days	0.45 ± 0.01 ^d^
18 days	0.49 ± 0.01 ^c^
21 days	0.33 ± 0.01 ^f^
25 days	0.38 ± 0.01 ^e^

A *p* value of <0.05 was considered to be statistically significant (indicated by superscript letters).

**Table 2:** Effect of diet on average daily weight gain. Data are expressed as mean ± SEM (*n* = 15).

Average Daily Gain (g/day)	7 Days	18 Days	25 Days
Melanoidin-free	0.17 ± 0.07 ^abcA^	0.19 ± 0.07 ^aA^	0.31 ± 0.07 ^abA^
25% Melanoidins	0.048 ± 0.07 ^cB^	0.34 ± 0.07 ^aA^	0.47 ± 0.07 ^aA^
50% Melanoidins	0.28 ± 0.07 ^abA^	0.18 ± 0.07 ^aA^	0.18 ± 0.07 ^bA^
75% Melanoidins	0.34 ± 0.07 ^aA^	0.20 ± 0.07 ^aAB^	0.14 ± 0.07 ^bB^
100% Melanoidins	0.13 ± 0.07 ^bcA^	0.19 ± 0.07 ^aA^	0.23 ± 0.07 ^bA^

Different letters indicate significant differences (*p* < 0.05; ANOVA). The lowercase letters indicate significant differences (*p* < 0.005; ANOVA) on the same day between different groups. The capital letters indicate significant differences (*p* < 0.05; ANOVA) in the same group between different days.

**Table 3:** Effects of melanoidin malts on short-chain fatty acids. Data are expressed as mean ± SEM (*n* = 15).

	Melanoidins	Day 0	Day 3	Day 7	Day 14	Day 21
Acetate (mmol/mL)	0%	4.01 ± 0.68 ^a^	3.1 ± 0.94 ^aA^	4.1 ± 1.33 ^aA^	2.35 ± 0.97 ^aA^	2.13 ± 0.63 ^aB^
	100%	4.01 ± 0.68 ^a^	3.7 ± 1.33 ^aA^	3.6 ± 1.41 ^aA^	4.14 ± 1.05 ^aA^	4.51 ± 0.43 ^aA^
Propionate (mmol/mL)	0%	0.25 ± 0.03 ^a^	0.21 ± 0.03 ^aA^	0.16 ± 0.04 ^aA^	0.07 ± 0.03 ^bA^	0.06 ± 0.01 ^bB^
	100%	0.25 ± 0.03 ^a^	0.14 ± 0.05 ^aA^	0.11 ± 0.04 ^bA^	0.09 ± 0.02 ^bA^	0.09 ± 0.03 ^bA^
Butyrate (mmol/mL)	0%	0.42 ± 0.17 ^a^	0.27 ± 0.04 ^aA^	0.18 ± 0.07 ^aA^	0.06 ± 0.03 ^bB^	0.10 ± 0.01 ^bA^
	100%	0.42 ± 0.17 ^a^	0.13 ± 0.04 ^bA^	0.09 ± 0.02 ^bA^	0.14 ± 0.01 ^bA^	0.08 ± 0.02 ^bA^
Total (mmol/mL)	0%	4.7 ± 0.88	3.6 ± 1.01	4.44 ± 1.44	2.5 ± 1.03	2.29 ± 0.62
	100%	4.7 ± 0.88	3.8 ± 1.42	3.8 ± 1.5	4.4 ± 1.08	4.68 ± 0.48

The same letters indicate no significant difference. Different letters indicate significant differences (*p* < 0.05; ANOVA). The small letters indicate significant differences (*p* < 0.005; ANOVA) with the same diet on different days. The capital letters indicate significant differences (*p* < 0.05; ANOVA) on the same day between different diets.

## Supplementary Materials

The following are available online at https://www.mdpi.com/2072-6643/12/1/241/s1, Figure S1: Impact of increasing dietary melanoidin malts on the composition of the gut microbiota (non-metric multidimensional scaling (NMDS) at Day 3 and 14, Figure S2: Temporal evolution of selected genera along the study and impact of malt consumption overall, Figure S3: Impact of different portions of dietary melanoidin malts on responsive genera abundances among the other phyla. (**A**) Firmicutes (ClostridiumXIVb), (**B**) (Dorea), (**C**) (Latobacillus); Bacteroidetes (**D**) (Alistipes), (**E**) Actinobacteria (Bifidobacterium), (**F**) Verrumicrobia (Akkermansia); (**G**) Proteobacteria (Parasutturella). Significant differences (*p* < 0.005) are indicated by different letters (*n* = 5).
