# Supplementary material for: Gut Microbiota Modulation by Dietary Barley Malt Melanoidins
Source: Nutrients. 2020 Jan 17;12(1):241. doi: 10.3390/nu12010241 (PMC7019678; doi:10.3390/nu12010241)
Supplement: Supplementary file 1 [file nutrients-12-00241-s001.pdf]

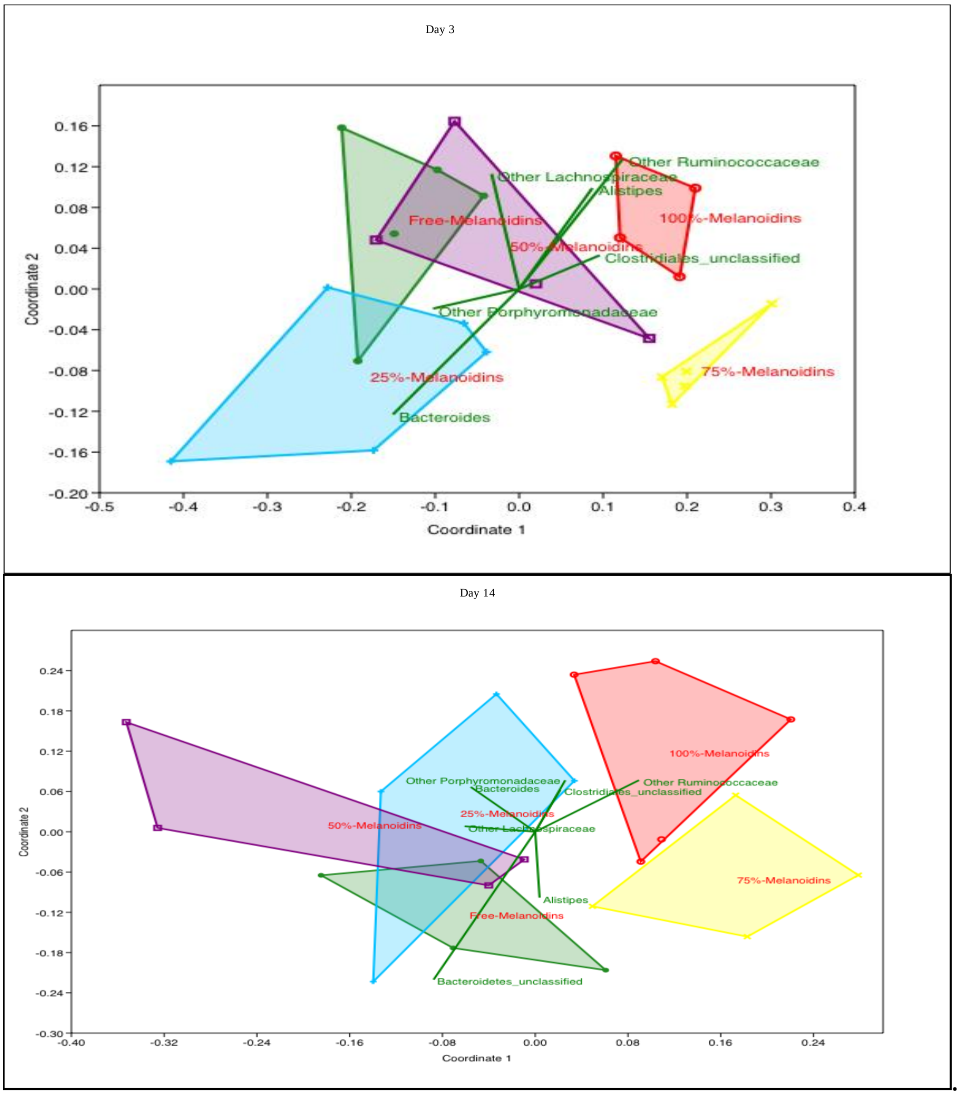


**Figure S1**: Impact of increasing dietary melanoidin malts on the composition of the gut microbiota (non-metric multidimensional scaling (NMDS) at Day 3 and 14.

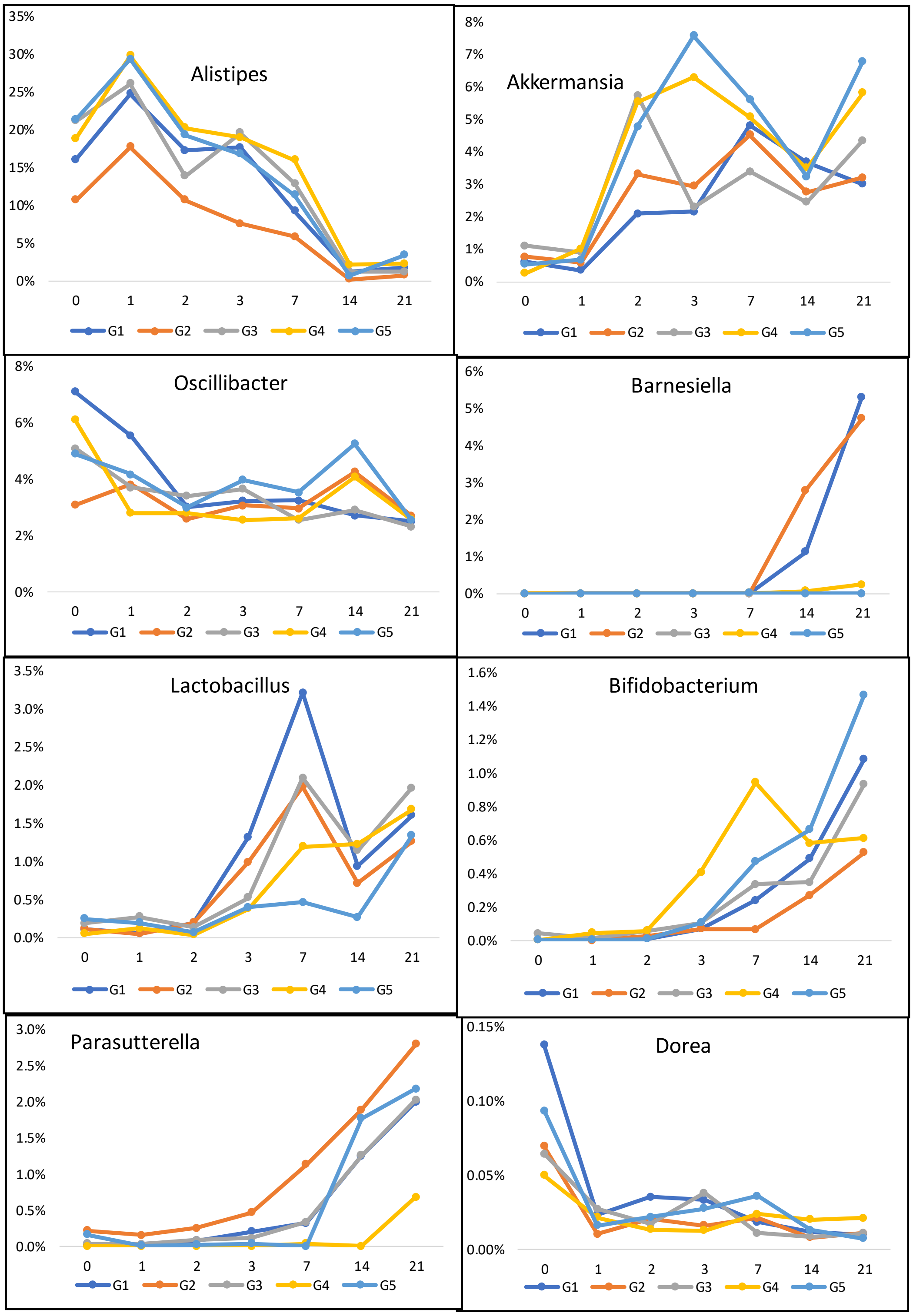


**Figure S2**: Temporal evolution of selected genera along the study and impact of malt consumption overall.

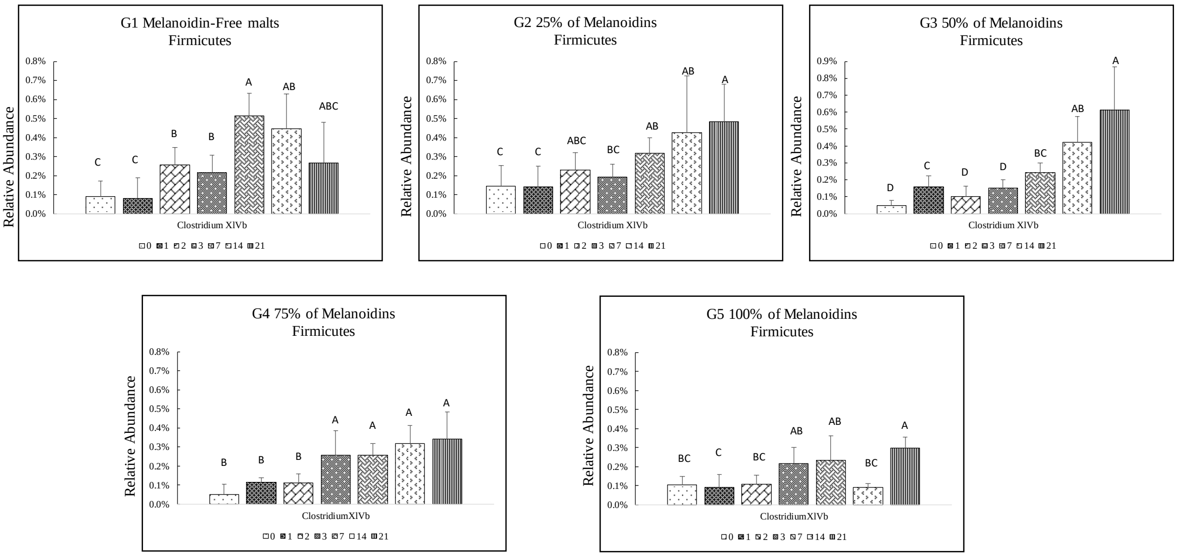
G1 Melanoidin-Free malts
Firmicutes
G2 25% of Melanoidins
Firmicutes
G3 50% of Melanoidins
Firmicutes
G4 75% of Melanoidins
Firmicutes
G5 100% of Melanoidins
Firmicutes
Relative Abundance
Clostridium XlVb
ClostridiumXlVb
0 1 2 3 7 14 21

**(A)**

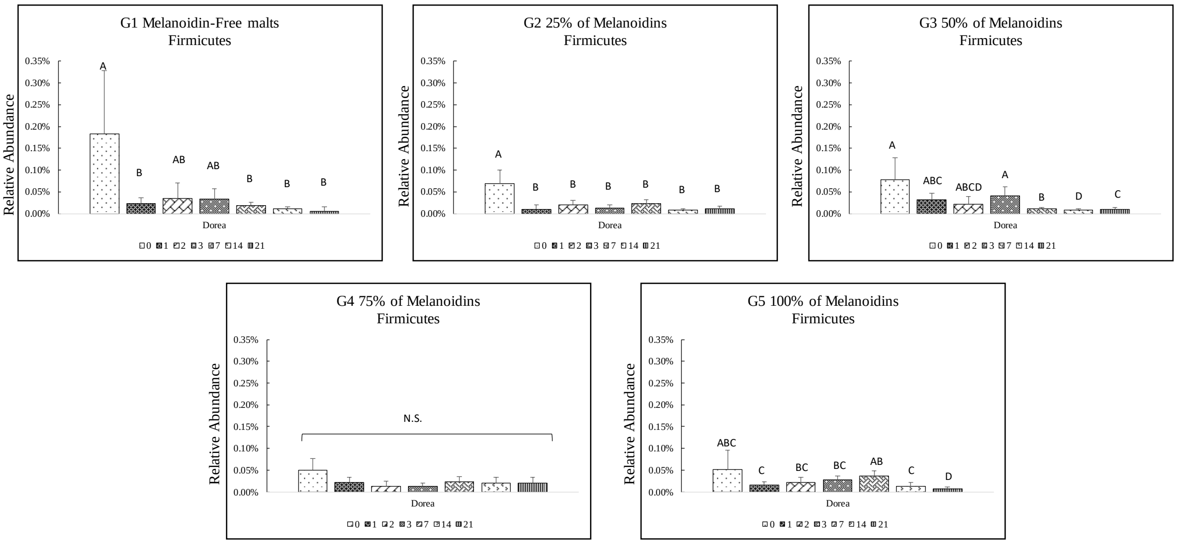
G1 Melanoidin-Free malts
Firmicutes
G2 25% of Melanoidins
Firmicutes
G3 50% of Melanoidins
Firmicutes
G4 75% of Melanoidins
Firmicutes
N.S.
G5 100% of Melanoidins
Firmicutes
Relative Abundance
Dorea
0 1 2 3 7 14 21

**(B)**

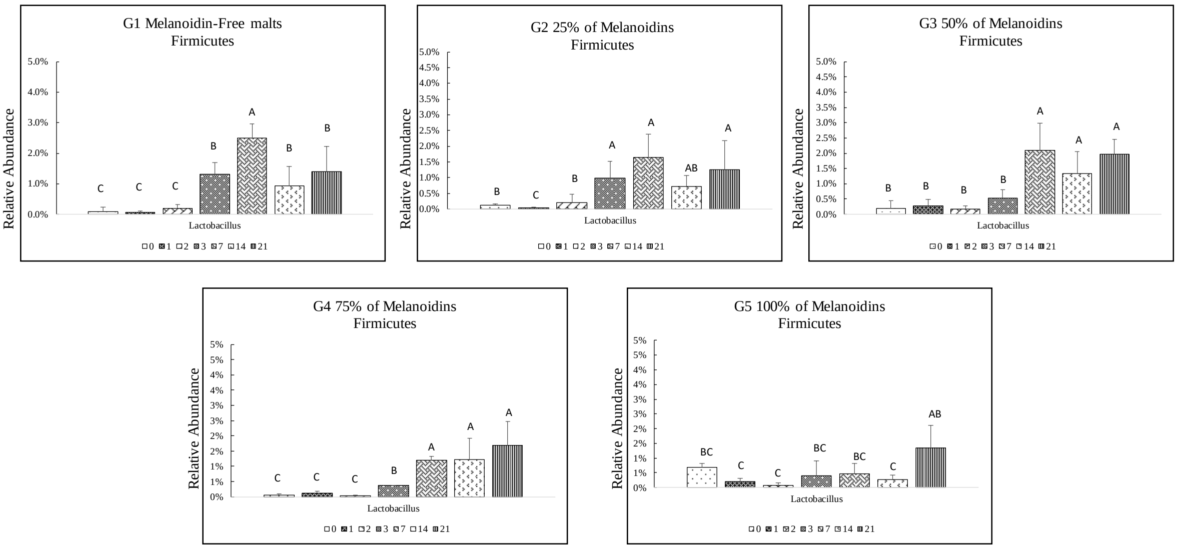
G1 Melanoidin-Free malts
Firmicutes
G2 25% of Melanoidins
Firmicutes
G3 50% of Melanoidins
Firmicutes
G4 75% of Melanoidins
Firmicutes
G5 100% of Melanoidins
Firmicutes
Relative Abundance
Lactobacillus
0 1 2 3 7 14 21

**(C)**

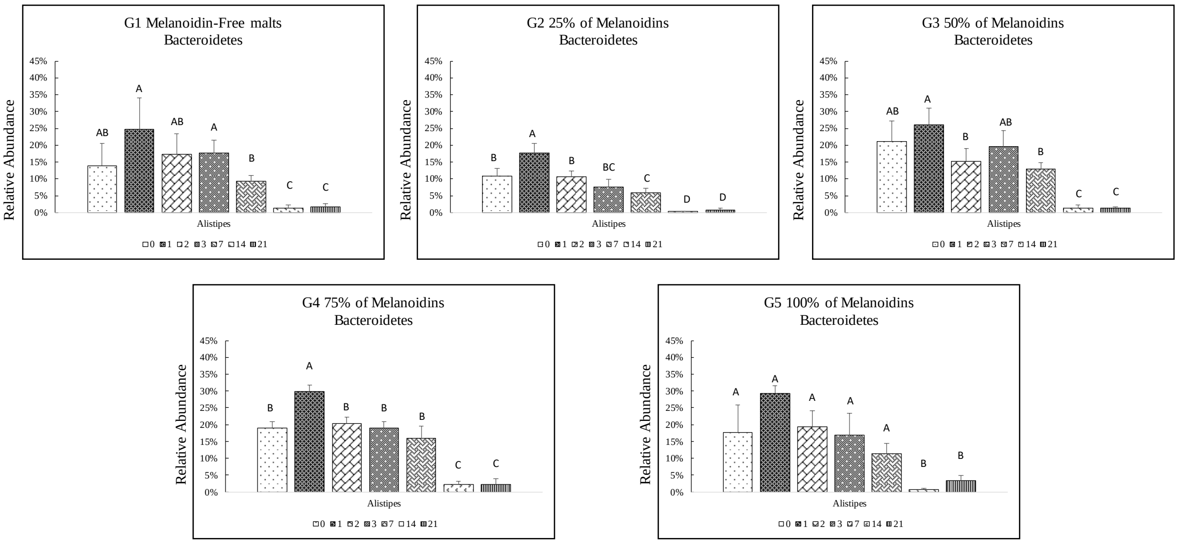
G1 Melanoidin-Free malts
Bacteroidetes
G2 25% of Melanoidins
Bacteroidetes
G3 50% of Melanoidins
Bacteroidetes
G4 75% of Melanoidins
Bacteroidetes
G5 100% of Melanoidins
Bacteroidetes
Relative Abundance
Alistipes
0 1 2 3 7 14 21

**(D)**

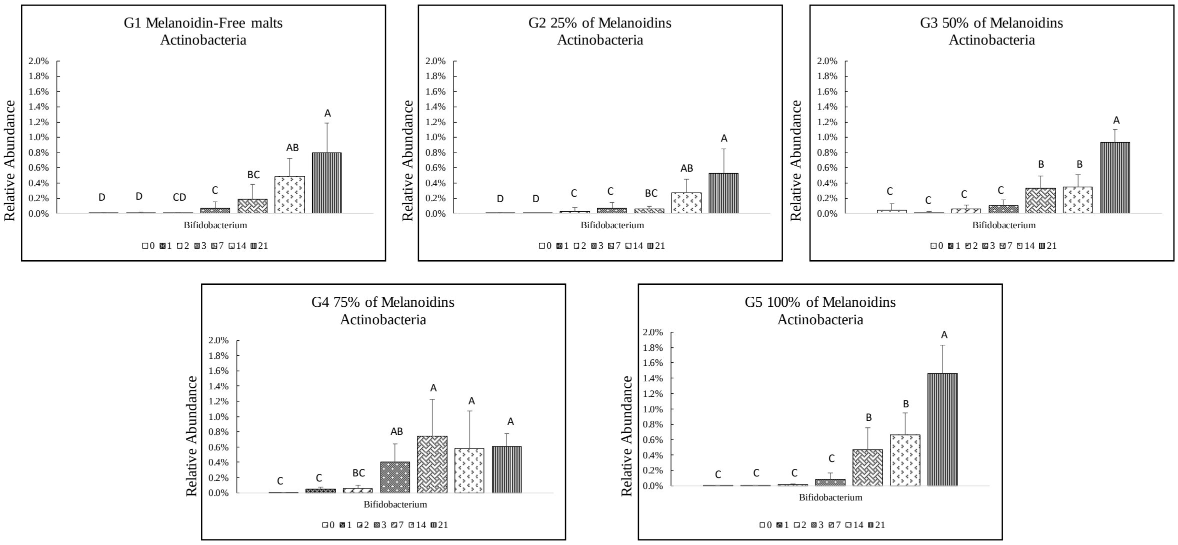
G1 Melanoidin-Free malts
Actinobacteria
G2 25% of Melanoidins
Actinobacteria
G3 50% of Melanoidins
Actinobacteria
G4 75% of Melanoidins
Actinobacteria
G5 100% of Melanoidins
Actinobacteria
Relative Abundance
Bifidobacterium
0 1 2 3 7 14 21

**(E)**

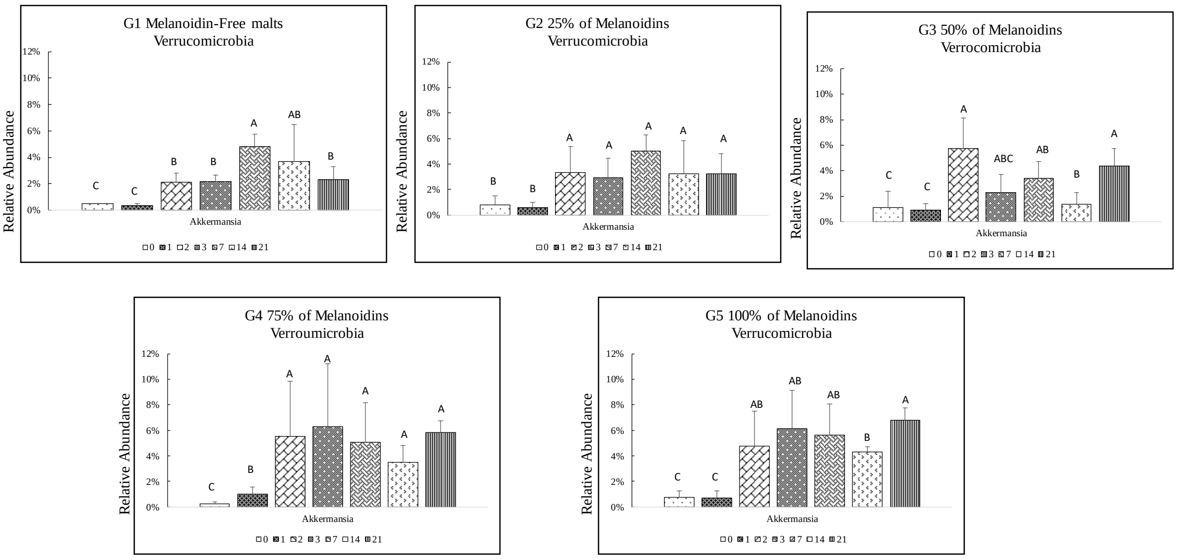
G1 Melanoidin-Free malts
Verrucomicrobia
G2 25% of Melanoidins
Verrucomicrobia
G3 50% of Melanoidins
Verrocomicrobia
G4 75% of Melanoidins
Verroumicrobia
G5 100% of Melanoidins
Verrucomicrobia
Relative Abundance
Akkermansia
0 1 2 3 7 14 21

**(F)**

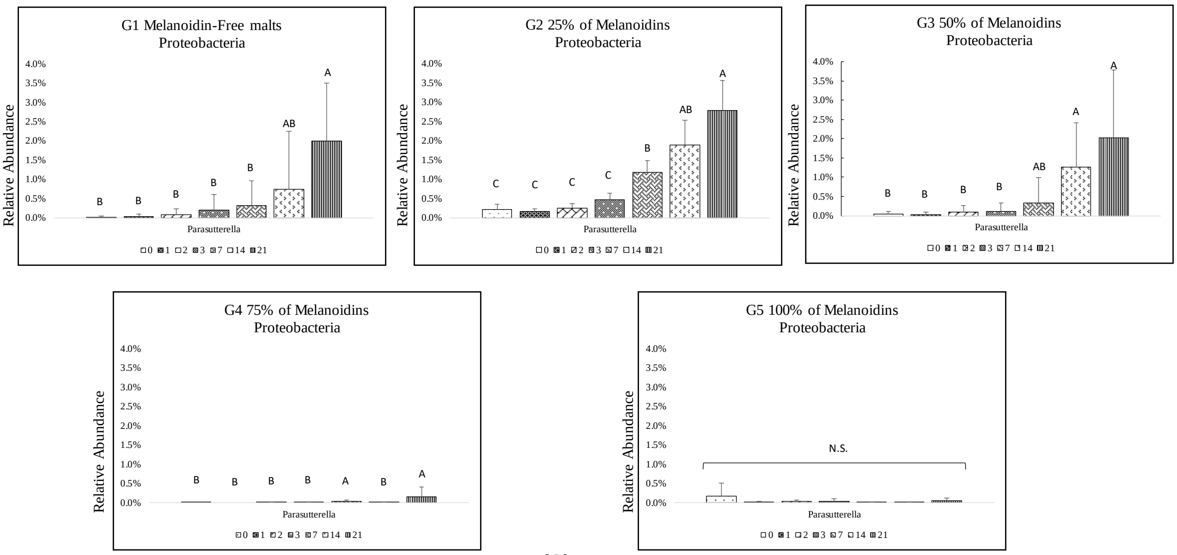


**(G)**

**Figure S3**: Impact of different portions of dietary melanoidin malts on responsive genera abundances among the other phyla. (**A**) Firmicutes (ClostridiumXIVb), (**B**) (Dorea), (**C**) (Latobacillus); Bacteroidetes (**D**) (Alistipes), (**E**) Actinobacteria (Bifidobacterium), (**F**) Verrumicrobia (Akkermansia); (**G**) Proteobacteria (Parasutturella). Significant differences ($p < 0.005$) are indicated by different letters ($n$ =5).
